# Resolving Barriers to Continence for Children with Disabilities: Steps Toward Evidence-Based Practice

**DOI:** 10.1007/s40617-023-00891-0

**Published:** 2023-12-26

**Authors:** Maeve G. Donnelly, Amanda M. Karsten

**Affiliations:** 1 Western New England University, Springfield, MA USA; 2 Northeastern University, Boston, MA USA; 3 Grand Valley State University, Allendale, MI USA

**Keywords:** Toilet training, Continence/incontinence, Autism spectrum disorder, Intellectual disability

## Abstract

Behavior-analytic toilet training (BATT) methods to support urine continence have been reviewed and replicated in numerous studies. Despite empirical validations of BATT, children with disabilities may not experience successful toilet training nor access the associated health and social benefits of urinary continence. It is possible these outcomes are partially due to practical barriers that arise throughout urine training. In practice, barriers may interfere with toilet training to the extent that training is postponed or discontinued, resulting in long-term incontinence and other related problems. Examples of barriers include problem behavior, excessive urine retention, recurrent accidents, and excessive or insufficient independent self-initiations to toilet. Researchers have sometimes described strategies to address these types of barriers. However, practitioners may not be aware of these strategies because they are secondary to the purpose of an investigation and may only apply to a subset of participants. The purpose of this review article is to synthesize the collection of barrier solutions described in published research on urine training for children with developmental disabilities. Results may assist practitioners in modifying BATT according to an evidence-based practice framework until their clients overcome barriers to achieve urine continence.

Independent toileting skills have demonstrated value for a high quality of life, leading to greater access in community settings as well as enhanced opportunities for independent living (Matson & LoVullo, 2009). Independent toileting may be associated with reduced child abuse (Luxem & Christophersen, 1994). Conversely, a lack of independent toileting skills has been associated with a negative impact on peer relationships (e.g., teasing; Leader et al., 2018). Ongoing accidents can also affect family members and other interested parties when a great deal of time is needed to clean up after accidents and wash soiled clothing (McCartney & Holden, 1981). It should be noted that parents of children who achieve continence (i.e., absence of accidents) relatively late (after 42 months of age) produce higher scores on parent stress indices (Blum et al., 2004). These factors may be compounded for parents of children with disabilities, who report more overall stress than parents of nondisabled children (Niemczyk et al., 2019). Some parents of children with disabilities who are not toilet-trained report more stress than parents of children with disabilities who are toilet-trained (Macias et al., 2006).

Given these reasons, toileting independence is likely to be an example of socially important behavior change that behavior analysts may pursue with the clients and families they serve. One framework for describing how a behavior analyst should design and deliver services related to toileting and other client goals is the process of evidence-based practice (EBP). Evidence-based practice is rooted in the field of medicine (Sackett et al., 1996). Slocum et al. (2014) defined the EBP of applied behavior analysis as the process by which behavior analysts design individualized services based on (1) their professional judgment (e.g., scope of competence, knowledge of legal and ethical requirements); (2) the best available evidence (e.g., research literature, practice-based evidence); and (3) client values and contextual variables (e.g., client and community preferences, resource constraints). This framework is helpful because it aligns closely with standards in the Behavior Analyst Certification Board’s *Ethics Code for Behavior Analysts* (e.g., Standards 2.10, 2.13, 2.14, 2.18, and 2.19; i.e., the *Code*; BACB, 2020), registering practitioners’ responsibility to engage in conditional problem solving as opposed to following a recipe-style approach. In the realm of toilet training, the standards of EBP call on behavior analysts to first select an initial intervention that is supported by research and seems like a good contextual fit for the client and service setting. Next, behavior analysts implement and evaluate the effects of that intervention. Finally, behavior analysts are responsible for systematically modifying intervention methods as needed until the client’s goal is achieved or an alternative approach is warranted. Practitioners weigh considerations related to evidence, client-contextual factors, and professional judgment at each stage of this EBP process.

An important early step in the EBP approach to toilet training is the ruling out of potential physiological and medical reasons for incontinence (i.e., absence of urinary control; Bernard-Bonnin, 2000). Such causes of incontinence may include conditions such as urinary tract infections or an ectopic ureter (Schmitt, 2004). Incontinence may result from neurological disorders that affect physical development (e.g., spina bifida; Price & Butler, 2001). Medication side effects can also contribute to incontinence (e.g., risperidone has been associated with incontinence; Agarwal, 2000). After medical and physiological factors have been ruled out, incontinence may be conceptualized as the absence of independence with specific toileting skills (e.g., holding urine between toilet visits, unprompted requesting or walking to the bathroom, removing clothing, eliminating, wiping, dressing, flushing, and washing hands). These skills are subject to the principles of behavior and may be acquired by establishing new behavior–environment relations.

The seminal behavior analytic toilet training package was developed by Azrin and Foxx (1971) for nine incontinent adults living in a state hospital. In addition to intellectual disabilities, most participants in the original study were noted to have physical disabilities or sensory impairments (e.g., partial paralysis or vision impairment) as well as minimal or no vocal-verbal communication. See Table 1 for a summary of Azrin and Foxx’s procedures. Independent toileting was observed after an average of six training days (range: 1–14 days) for all participants. Five months after training, accidents were “virtually absent” (p. 95). Foxx and Azrin (1973a) evaluated modified procedures (including dry pants checks, which consisted of guiding participants to use their hands to feel their pants and reinforcing accurate confirmation of dry pants) with 34 typically developing children. They later published two books detailing this technology (*Toilet Training Persons with Developmental Disabilities*, Foxx & Azrin, 1973b; *Toilet Training in Less Than a Day*, Azrin & Foxx, 1989).

**Table 1:** Components of behavior-analytic toilet training as described by Azrin and Foxx (1971)

Component	Description
Immediate detection of elimination	An alarm was affixed to each participant’s clothing to alert the trainer to the occurrence of an accident; a second alarm was placed inside the toilet bowl to signal continent urination and permit contingent reinforcement
Prevent behaviors incompatible with toileting	Participants remained seated within 1 m of the toilet throughout the 8-hr session except when consuming lunch; the area around the toilet was partitioned to minimize access to other visual stimuli
Increase the frequency of urination	Large quantities of fluid were presented each half-hour to maximize opportunities for differential consequences for accidents and continent urination
Stimulus control of elimination response	Participants were prompted to sit on the toilet for 20 min of every 30-min period to increase the likelihood that urination would occur in the toilet and eventually be occasioned by sitting on the toilet
Positive reinforcement of correct toileting	Continent urination was followed by preferred edibles, praise, and hugs. Preferred edibles were delivered after 5 min in the absence of accidents
Dressing skills training	Least-to-most physical prompting was used to have the participants undress and re-dress at each scheduled and self-initiated toileting opportunity
Independent toilet approach (self-initiation of toileting)	Trainers used the minimal effective prompt for toileting and gradually faded that prompt until it was eliminated
Modeling	Multiple participants were trained at the same time; chairs were placed close together in close proximity to toilets to facilitate participant-participant observation
Inhibition of “accidents”	Following each accident, participants were reprimanded and prompted to engage in cleanliness training (consisting of showering, changing clothes, rinsing out soiled clothes and hanging them to dry); a 1-hr timeout from fluids, foods, and social interaction was implemented

Since the publication of Azrin and Foxx’s studies and books, researchers have assessed the efficacy of variations of this package to address urinary incontinence across a variety of populations, including children with autism spectrum disorder (ASD) and other disabilities. In their review of the toilet training literature, Kroeger and Sorensen-Burnworth (2009) refer to such procedures as “rapid toilet training.” However, “rapid” is a relative term. For example, Johnson et al. (2021) reviewed the literature and found that total toilet training hours ranged from 17.5 hr to 1,848 hr across studies and participants, with a mean total duration of 373.3 hr. Other researchers have described their toilet training packages as “Intensive Toilet Training” (e.g., Cagliani et al., 2021) or “Modified Intensive Toilet Training Method” (e.g., Spencer & Ello., 2021). Components of each package vary, but these interventions are identifiable by their conceptual basis in the principles of behavior as well as their inclusion of several, if not all, components of the seminal Azrin and Foxx (1971) procedure. Throughout this article, interventions based on the Azrin and Foxx procedure will be referred to as “Behavior-Analytic Toilet Training” (BATT).

Despite evidence supporting the efficacy of BATT garnered over the last 50 years, children with disabilities have shown a relatively high rate of incontinence throughout that same period. Williams et al. (2003) reported parent responses to questions about 102 children with ASD and 106 children with other developmental disabilities. From their sample, 45% of parents of children with ASD were concerned about their child’s toileting, and parents of 19% of children with other developmental disabilities reported such concerns. Von Gontard et al. (2015) found that children with ASD showed increased rates of daytime urinary incontinence (25%) when compared to age-matched peers without ASD (approximately 5%). Leader et al. (2018) identified toileting issues for children with ASD in their survey of 127 children and adolescents with ASD living in at least five different countries, noting that toileting problems were significantly greater among children diagnosed with both ASD and an intellectual disability. Likewise, Berry-Kravis et al. (2019) observed that receiving an ASD diagnosis is a predictor of delayed bladder training. Toileting problems may continue into adulthood: Henderson et al. (2009) found that approximately 30% of 1373 adults between 40 and 89 years of age with intellectual or developmental disabilities displayed toileting deficits.

One factor potentially contributing to ongoing toileting concerns among people with disabilities is the range of barriers that may arise and prevent the effective application of BATT. For example, in an analysis of the results of 44 case studies, Lomas Mevers et al. (2018) noted that over 45% of participants required the use of modified strategies to address barriers. It is reasonable to conclude that practitioners also encounter barriers with their clients (e.g., behavior that prevents completion of toileting routines or recurring accidents even after continent voids are established). Some researchers have developed individualized strategies for surmounting barriers to independent toileting. These strategies are often unanticipated and secondary to the original research question, making information about them less accessible for practitioners who encounter similar situations.

It is important to recognize that the term “continence” (i.e., absence of accidents) refers to both urine and bowel continence. However, urine and bowel training have been described as different stages of toilet training, and these stages can be further broken down to address daytime and nighttime incontinence (e.g., Burns & Matson, 2017). Studies comprising the toilet training literature do not typically address all stages within a single article; rather, they often focus on daytime urine continence (e.g., Perez et al., 2021). Lauters et al. (2022) recommend addressing daytime urine continence prior to resolving nighttime incontinence. Thus, the purpose of this review and discussion is to name barriers to daytime urine training for children with developmental disabilities observed in the experimental research on BATT and describe individualized solutions to these barriers developed by researchers. The resulting collection of strategies may assist practitioners as they engage in the process of EBP on behalf of children with developmental disabilities who do not achieve urinary continence with initial variations of BATT.

## Method

### Search and Review Procedures

To conduct this search, we replicated the method used by Kroeger and Sorensen-Burnworth (2009) in their review of published toilet training protocols. The first author searched the PsycInfo, Medline, and ERIC library databases for all possible combinations of the following terms: “toileting, toilet, continence, and incontinence” and “autism, developmental disability and disorder, intellectual disability, and mental retardation.” We limited publication years to 1971–2023 to coincide with the publication of Azrin and Foxx (1971), and we selected academic journal publications only. In contrast to Kroeger and Sorensen-Burnworth, criteria for relevance to this review included a description of initial failure to toilet train in the presence of at least three of the BATT components listed in Table 1 as well as subsequent modification(s) to BATT for at least one participant.

As a first step in the selection process, articles that were not available in English were eliminated. Next, articles unrelated to the topics of daytime urine training children with disabilities or problematic toileting-related behaviors that interfered with toileting were discarded. Lastly, studies of interventions other than BATT or its components (e.g., solely pharmacological interventions) were eliminated. The full texts of all remaining articles were reviewed by the first author, and the reference lists of these articles were examined to identify additional applicable studies not returned from the database searches. Articles that solely referenced interventions outside of the scope of the *Code* (e.g., exclusive use of aversive stimuli; BACB, 2020) were discarded. Articles describing the results of participants who did not have any documented exposure to at least three BATT components (as noted in the participant summary, during baseline, or during a first intervention phase) prior to the BATT modification being implemented in the study were excluded. This qualification ensured that the modification was necessitated due to a barrier arising during training which prevented the acquisition of continence. One exception to this criterion applied to articles in which participants previously achieved urine continence but engaged in behaviors that were incompatible with the toileting routine (e.g., destruction of toileting environments) or that suggested additional functions of toileting behavior (e.g., urine accidents maintained by social negative reinforcement). Articles that described BATT interventions that proceeded as planned in the absence of any barriers were set aside. As a final step, articles that described potential barriers present prior to the initiation of daytime urine training (e.g., sensory impairments) or after the completion of training (e.g., ongoing overnight urinary incontinence) were set aside.

### Intercoder Agreement

After the first author identified the pool of articles meeting the criteria specified above, an undergraduate research assistant independently reviewed all articles for the presence of specific barriers by comparing the barrier definitions listed in Table 2 with the content described in the methods and results sections of each study. Intercoder agreement for the presence of barriers across all studies was 84.3% (43/51). This percentage was obtained by dividing the number of agreements that a particular barrier was present in a given study by the sum of agreements plus disagreements, and then multiplying by 100. Disagreements reflected instances in which one coder judged the presence of a particular barrier in a given study but the other coder did not observe that barrier. Articles were also coded in terms of study characteristics (research design and number of participants). The second author coded 25% of articles for research design, and 100% of articles were coded for number of participants by the undergraduate research assistant. Interobserver agreement of coded study characteristics was 100%.

**Table 2:** Description of barriers to toilet training

Barriers to Toilet Training	Potential Range of Topographies
Problem Behavior	Aggressive responding, self-injurious behavior, environmental destruction, excessive flushing, water play, sexualized behavior, socially maintained urination away from the toilet
Excessive Urine Retention	Absence of continent urination during toilet training; urination occurring only when the child is wearing a diaper or in clothing away from the toilet, absence of urination during toilet sits or throughout a session; partial, rather than full, emptying of the bladder during a toilet sit
Recurrent Accidents	Ongoing accidents despite consistent continent urination, misdirected urination
Problems with Self-Initiations	Excessive self-initiations that do not appear to be occasioned by a full bladder or maintained solely by relief of a full bladder, absence of independent self-initiations, decrease in self-initiations

## Results

The keyword search returned 923 initial results, 784 of which were academic journal articles. When articles not available in English were set aside, 729 articles remained. Duplicates occurring across the three databases were removed, leaving 556 articles. Following the examination of abstracts for the above-mentioned criteria as well as full-text review of apparently applicable articles, a total of 22 studies were identified as meeting the criteria for inclusion. Four additional articles were identified through review of the reference lists of these articles and corresponding abstract and/or full-text review. All 26 identified articles were categorized according to the type(s) of barrier described. Readers should note that the barrier categories named in this review were arrived at inductively and do not always reflect the exact terms that primary source authors used to describe each barrier. Further, few studies employed experimental methods to evaluate modifications, and primary source authors did not refer to modifications as “solutions.” To facilitate readers’ identification of information with relevance to their clients or applied research questions, results are organized by the order in which these barriers may arise during a toilet training case.

Four categories of barriers to daytime urine continence were identified: persistent problem behavior occurring in the toileting setting, excessive urine retention (i.e., withholding urine even while sitting on the toilet), recurrent accidents (i.e., ongoing accidents despite experiences with reinforcement for continent voids), and problems with self-initiations to toilet. Table 2 shows the range of topographies identified within each barrier category. For example, the barrier category “problems with self-initiations” includes both excessive self-initiations and absence of self-initiations. Table 3 shows the number of studies addressing each barrier category ordered by date of publication. Sixteen studies corresponded to multiple barrier categories. Eleven studies described problem behavior that occurred during toilet training, and 10 of those studies described modifications related to the problem behavior that occurred. Withholding urination during training was observed in 13 studies and addressed with a procedural modification in 11 of those identified studies. Recurrent accidents were observed in 17 studies and addressed in 10 of these studies. Five of the 11 studies that described problems with self-initiations attempted solutions to that barrier.

**Table 3:** Literature indicating barriers to toilet training in children with disabilities

Studies				
	Problem Behavior	Excessive Urine Retention	Recurrent Accidents	Problems with Self-Initiations
Siegel (1977)			**♦♦**	
Richmond (1983)			**♦♦**	
Luiselli (1987)			**♦♦**	**♦♦**
Heyward (1988)		**♦♦**		
Hagopian et al. (1993)	**♦♦**	**♦♦**	♦	
Taylor et al. (1994)		**♦♦**		
Luiselli (1996b)		**♦♦**	♦	
Luiselli (1997)		**♦♦**		
Duker et al. (2001)	**♦♦**		**♦♦**	
Ewing et al. (2001)				**♦♦**
Cicero & Pfadt (2002)				**♦♦**
Ricciardi & Luiselli (2003)	♦		**♦♦**	**♦♦**
Post & Kirkpatrick (2004)	**♦♦**	♦	♦	
LeBlanc et al. (2005)		**♦♦**	♦	♦
Chung (2007)	**♦♦**		**♦♦**	
Foxx & Garito (2007)	**♦♦**		**♦**	
Luiselli (2007)		**♦♦**		
Brown & Peace (2011)	**♦♦**	♦		♦
Cocchiola et al. (2012)			**♦♦**	
Doan & Toussaint (2016)	**♦♦**		♦	♦
Flood & Luiselli (2016)	**♦♦**	**♦♦**		
Lomas Mevers et al. (2018)	**♦♦**	**♦♦**	**♦♦**	**♦♦**
Perez et al. (2020)		**♦♦**	**♦♦**	♦
Dowdy et al. (2020)	**♦♦**			
Cagliani et al. (2021)			**♦♦**	♦
Croteau et al. (2022)		**♦♦**	♦	♦

♦♦ indicates that a modification was implemented to address the named barrier within the given study, whereas ♦ indicates that the barrier was observed but not addressed with a specific modification. Additional supporting studies that did not meet inclusion criteria for this review are cited in text

Results are summarized with an introductory description of each barrier followed by a description of the potential solutions found in the studies included in this review. Results are also displayed in Table 4.

**Table 4:** Solutions for barriers to toilet training described in research

Barriers to Toilet Training	Solutions	Examples
Problem Behavior	1. Modify or eliminate potentially aversive treatment components	A. Discontinue overcorrection* B. Apply a less restrictive or simplified version of overcorrection* C. Allow the child to avoid full overcorrection contingent upon continent urination as a correction response D. Decrease the duration of exposure to toileting demands or aversive stimuli (e.g., the toilet)*
	2. Modify access to stimuli	A. Discontinue or limit activities such as self-cleaning if they reliably precede problem behavior B. Provide noncontingent access to preferred stimuli* C. Conduct a competing items assessment to identify stimuli that may compete with problem behavior in the toileting setting and provide those stimuli noncontingently
	3. Implement functional assessment and differential reinforcement	A. Conduct functional assessment of interfering problem behavior and provide reinforcers contingent on alternative responses* B. Conduct functional assessment of accidents (urination occurring away from the toilet)* C. Arrange the environment to support extinction for socially maintained urination D. Reduce overall demands when accidents appear to be maintained by escape from demands E. Use differential reinforcement to strengthen continent urination
Excessive Urine Retention	1. Implement procedures to transfer stimulus control of urination to the toilet	A. Shape urination in a diaper during training times or in the training environment; prompt the child to void on the toilet in a diaper, progressively cut the diaper away to fade* B. Shape daytime urination by reinforcing urination in the diaper at certain times of day C. Use a water prompt to elicit urination in the toilet; provide reinforcement contingent on continent voids D. Apply a percentile schedule of reinforcement to shape differentially longer voids E. Ensure that reinforcer delivery does not interrupt the flow of urine before it is complete
	2. Apply a negative reinforcement contingency for toilet sits	A. Allow the toileting session to end as soon as the child urinates in the toilet* B. End toilet training for the day following a single instance of urination in the toilet
	3. Increase opportunities for positive reinforcement of continent voids	A. Promote adequate fluid intake by offering preferred drinking containers B. Schedule toileting sessions to occur at the end of the day when the bladder is likely to be full; reinforce continent voids
Recurrent Accidents	1. Incorporate materials that enhance the salience of accidents	A. Use a urine alarm* B. Ensure children are wearing underpants* C. Conduct dry pants checks*
	2. Increase the frequency of scheduled toilet sits	A. Reduce the duration of the daily inter-sit interval to 15 min or less; fade the sit schedule throughout training* B. Collect data on the timing of accidents and shorten the inter-sit interval during times when accidents are regularly observed* C. Add a single additional sit during a common accident time
	3. Monitor fluid intake	A. Track fluid intake and observe for possible correlations with increased fluid and increased accidents* B. Discontinue fluid loading; reduce fluid intake to baseline levels
	4. Use a urine target	A. Provide a target for children to aim their urine stream toward; instruct children to urinate in the toilet
Problems with Self-Initiations	1. Teach children to respond to supplementary cues	A. Prompt children to toilet at certain times; direct the child to use a supplementary stimulus (e.g., a wristwatch) to manage their own toileting
	1. Discontinue toileting prompts to allow for self-initiations to emerge	A. Withdraw prompts to toilet and watch for emerging self-initiation skills as participants contact motivating operations for toileting*
	2. Identify the reinforcer for excessive requests	A. Assess correspondence between self-initiations and subsequent continent urination B. Conduct a functional analysis to identify the existing request–reinforcer relation
	3. Identify and fade unnecessary training components	A. Discontinue additional fluids, thin or remove the reinforcement schedule for continent voids, reduce time spent in the bathroom environment
	4. Implement a multiple schedule	A. Use discriminative stimuli to signal when self-initiated requests will be honored and when they will not be honored

* = reported across at least two studies within the literature that met criteria for this review

### How Can Practitioners Modify BATT for Children Who Engage in Problem Behavior?

Past research demonstrates that problem behavior may occur during BATT. Some studies have described generalized problem behavior that may occur across contexts such as aggressive responses or flopping, and others have described problem behavior that seems specific to the toileting environment. For example, Dalrymple and Ruble (1992) shared parent reports of disruptive behavior such as urinating out of the toilet, smearing feces, clogging the toilet with excessive amounts of toilet paper, and repeated unnecessary flushing. Strategies used to address problem behavior occurring during BATT are described below.

#### Modify or Eliminate Potentially Aversive Treatment Components

Some elements of BATT may constitute aversive stimulation for some individuals and thus occasion problem behavior. For example, longer-duration toilet sits increase the likelihood that a practitioner will observe and reinforce continent urination; however, problem behavior may also occur during extended sits due to either aversive stimulation or lack of access to alternative reinforcing activities. Overcorrection is a behavior-change strategy in which the child is required to engage in an effortful response that repairs an antecedent undesirable behavior (Cooper et al., 2020). Restitutional overcorrection, included in the Azrin and Foxx (1971) protocol, consists of requiring the participant to return the environment to an undisturbed or improved state (e.g., cleaning the floor after an accident). Positive practice overcorrection, included in Foxx and Azrin’s (1973b) procedure, consists of the participant repeating a desirable behavior multiple times following the undesirable behavior (e.g., walking to the bathroom and pulling their pants down and up multiple times following an accident). Overcorrection may occasion problem behavior, rendering BATT procedures that include overcorrection components impractical (Cicero & Pfadt, 2002).

##### Discontinue Overcorrection

Two studies eliminated overcorrection to reduce problem behavior. Doan and Toussaint (2016) noted distress in one participant during overcorrection in their evaluation of parent-implemented BATT. Overcorrection was discontinued by the parent to avoid further distress. Hagopian et al. (1993) observed self-injurious behavior (SIB) during toilet sits. Investigators discontinued overcorrection and implemented a differential reinforcement of other behavior contingency as well as reinforcement of continent urination. Investigators also reduced the duration of required toilet sits and blocked instances of SIB. Although these modifications to BATT resulted in decreases in SIB, decreases were also observed in continent voids. In other words, without overcorrection, the training package described by Hagopian et al. was safer but less effective for toilet training.

##### Modify Overcorrection

Two studies reported successful resolution of problem behavior when overcorrection was simplified rather than eliminated. Brown and Peace (2011) stated concerns related to aggressive and sexualized behavior (i.e., masturbation) occurring during changing of clothing and following directions in baseline. They implemented a simplified positive practice procedure consisting of a neutral verbal statement describing the accident and a single trip to the changing area where the participant was prompted to remove soiled clothing, wash, and dress. Investigators also modified the typical BATT toileting schedule such that toilet visits occurred only every 60–90 min. Near-zero rates of aggression and sexualized behavior were reported. Other examples of simplified procedures are evident in the literature: Richmond (1983) required participants to simply wash off and change clothes following accidents. Likewise, Cocchiola et al. (2012) stated, “You wet your pants. You need to change,” before guiding participants to the bathroom and assisting them in changing clothes following accidents without additional consequences. LeBlanc et al. (2005) stated, “No wet pants,” and required participants to complete a 1-min toilet sit immediately following accidents. Full positive practice was avoided if the participant urinated in the toilet during that toilet sit. Hanney et al. (2013) suggested that, if positive practice is implemented in this way (i.e., with an option to avoid repeated toilet visits contingent upon continent urination), children will experience fewer instances of full positive practice. Comparative studies have yet to evaluate the efficacy of partial positive practice.

##### Reduce the Duration of Toilet Sits

Three studies reported successful resolution of problem behavior when toilet sits were abbreviated. Like Hagopian et al. (1993), Post and Kirkpatrick (2004) reduced the prescribed duration of toilet sits in an effort to decrease problem behavior. At first, the investigators prescribed 20-min toilet sits following the observation that the participant urinated soon after shorter toilet sits had ended. However, the participant engaged in hitting and pinching during 20-min toilet sits. Investigators verbally redirected aggression and reduced the toilet sit duration to 10 min. Aggression decreased, and 80% of urination occurred in the toilet across a 3-day period, at which point investigators further reduced the duration of toilet sits to a maximum of 5 min without urination. Even a small reduction in toilet sit duration may be effective: Doan and Toussaint (2016) reduced sits from 5 to 3 min, and that change correlated with decreases in a participant’s crying behavior. This individualized modification was informed by the observation that voids tended to occur within the first 3 min of sitting on the toilet.

#### Modify Access to Stimuli

Fully independent toileting requires unassisted access to a toilet, toilet paper, soap, and water as well as dressing and undressing. In the training setting, access to these stimuli and activities may interfere with skill acquisition. On the other hand, providing access to preferred stimuli unrelated to training may enrich the environment and reduce interfering behavior. Studies describing these manipulations are shared below.

##### Limit Access to Stimuli that Occasion Problem Behavior

As noted above, Brown and Peace (2011) described masturbation that occurred during post-accident clothing changes, which can become problematic in public settings. In addition to limiting overcorrection, Brown and Peace omitted activities historically correlated with this behavior (e.g., unnecessary washing of the genital area) while increasing and reinforcing the participant’s independence in his toileting routine (i.e., differential reinforcement of alternative behavior; DRA), potentially contributing to reported decreases in masturbation. The specific contributions of each component in this training package are unknown; however, this strategy may also be useful for preventing the sort of interfering behavior described by Dalrymple and Ruble (1992; e.g., clogging the toilet may be avoided by limiting client access to excessive toilet paper).

##### Provide Noncontingent Access to Preferred Stimuli

Two studies reported attempts to resolve problem behavior with noncontingent access to preferred stimuli. Dowdy et al. (2020) provided noncontingent access to a cell phone that was demonstrated to compete with toilet lid destruction during routine bathroom visits. Results indicated a decrease in destruction from an average of 1.5 responses per minute to an average of 0.1 responses per minute. Although this behavior was targeted in part because it interfered with toileting routines, data on continent voids were not reported. Lomas Mevers et al. (2018) described noncontingent access to preferred items for three participants who engaged in problem behavior. It should be noted that these items were identified via preference assessment as being less preferred than the reinforcer delivered for continence behaviors. All three participants were noted to be continent during a follow-up assessment, but data were not reported for problem behavior.

#### Implement Functional Assessment and Differential Reinforcement

Research indicates the use of functional assessment to identify reinforcers for interfering problem behavior such as property destruction, but urination away from the toilet can also acquire a socially mediated function. In these cases, inappropriate urination or socially maintained urination may be apt terms because the term accident implies a lack of physical control or skill. Discerning whether urination away from the toilet is evidence of a skill deficit or a history of differential consequences requires assessment of the conditions under which accidents occur. Socially maintained urination may be suspected when accidents are temporally related to previously established escape-maintained topographies of behavior (e.g., vocal refusal, destroying work materials) or when children reliably pull down their pants prior to urinating away from the toilet. In either case, functional assessment remains a useful tool for informing differential reinforcement contingencies to increase functional communication and continent urination. Although differential reinforcement is embedded in BATT (i.e., self-initiations and continent urination are differentially reinforced), some studies detail the use of additional strategies to address problem behavior.

#### Assessment of Interfering Problem Behavior

Two studies reported assessment-informed solutions for problem behavior. Flood and Luiselli (2016) described an intervention targeting crying and yelling during bathroom visits and avoidance of community bathrooms. Functional assessment results suggested that crying and yelling were maintained by negative reinforcement in the form of escaping or avoiding the presence of other people near the bathroom. Investigators provided tokens contingent on “talking nicely” (i.e., the absence of crying and yelling in the bathroom) while therapist proximity was systematically increased. Tokens could be exchanged for access to video games. This contingency was successful in increasing toleration of others near the bathroom. Likewise, Dowdy et al. (2020; described above) implemented functional assessment of toilet lid destruction as well as a competing stimulus assessment to inform their noncontingent reinforcement intervention for this automatically maintained behavior.

#### Assessment of Accidents

Two studies identified social consequences as potential maintaining variables for accidents. Ricciardi and Luiselli (2003) conducted functional assessment interviews and observations before concluding that urination away from the toilet was maintained by escape from demands and access to attention for a participant who also demonstrated independent toileting. During the intervention, investigators provided differential attention for continent urination as well as escape extinction (i.e., accidents were no longer followed by escape from demands). They required the participant to wear a diaper during escape extinction so that urination would not require an immediate trip to the bathroom or change of clothes. Accidents resolved, and diapers were discontinued. Antecedent manipulations may also be helpful in these situations: Foxx and Garito (2007) reduced overall instructional time from 6 hr per day to 2.5 hr per day after observing that urinary and bowel movement accidents appeared to be maintained by escape from demands.

### How Can Practitioners Modify BATT for Children Who Withhold Urination on the Toilet?

Behavior analytic toilet training typically includes frequent, extended-duration toilet sits and increased fluid consumption across long (e.g., 8-hr) training periods. Whereas these procedures often increase the negative reinforcing value of urinating in the toilet, studies have included some children who hold their urine or who produce very small amounts of urine while withholding proportionally larger amounts, increasing the likelihood of accidents later in the day. Unlike other toileting responses (e.g., sitting, wiping), urination cannot be manually prompted by the trainer. When BATT does not result in continent voids and resulting opportunities for reinforcement, research indicates the use of individualized strategies exemplified in the following studies.

#### Transfer Stimulus Control to the Target Conditions for Toileting

A case of faulty stimulus control may be suspected when children withhold urination and urinate only at night or when wearing a diaper outside of BATT sessions. Two potential solutions have been described for establishing urination discriminated by the presence of the toilet and associated bathroom stimuli.

##### Employ Shaping and Fading Techniques

Luiselli (1996a) designed a procedure to transfer stimulus control over urination from the diaper to the toilet. He recommended that children wear training pants except during training sessions (scheduled every 60–90 min), when they should wear a diaper and sit on the toilet. After 3–5 min of sitting on the toilet, the diaper should be removed, and reinforcement delivered if urination has occurred. Luiselli recommended that stimulus fading, consisting of cutting a 1-in hole in the diaper, should begin after the child wets the diaper in 85%–100% of training sessions for 1 week. The hole should be made progressively larger until continent urination occurs consistently.

Three studies implemented this diaper fading procedure when children did not urinate in the toilet. Luiselli (1996b) initiated diaper fading with a participant who only urinated in disposable diapers, despite repeated caregiver attempts to prompt continent urination. After the participant was reliably urinating on the toilet while wearing a diaper, she began to sit on the toilet independently before the altered diaper could be applied during training sessions. In this case, simply reinforcing urination that occurred while the participant was sitting on the toilet while wearing a diaper appeared to be sufficient to transfer stimulus control. Lomas Mevers et al. (2018) reported that a similar diaper fading procedure was associated with continence at a follow-up assessment within 6 months of training for two participants in their study who experienced this modification. Diaper fading requires that voids regularly occur during daytime hours (or whenever training is possible). Therefore, it may be necessary to shape daytime urination prior to implementing this modification. Smith et al. (2000) demonstrated this process by reinforcing urination in the diaper at certain times of day. After several days, Smith et al. introduced a chair, which the participant sat upon in a diaper to eliminate before moving to the toilet and undergoing successful diaper fading.

Stimulus control manipulations may also be useful if the child does not wear diapers. Despite long scheduled toilet sits (upwards of 15 min), Taylor et al. (1994) observed that their participant typically had an accident within 3 min of dressing after a toilet sit. The investigators hypothesized that underpants were functioning as a discriminative stimulus for urination and suspected that the tactile differences between clothing and the toilet may have inhibited urination. To transfer stimulus control from underpants to the toilet, Taylor et al. removed the participant’s pants and underpants 5 min after delivering liquids. The participant sat on the toilet unclothed until 10 min had passed or he had urinated in the toilet, whichever occurred first. The duration of time per session that the participant was undressed was faded based on the occurrence of continent urination. Zero accidents were reported at 10-month follow-up observations, and unprompted continent urination was reported to occur approximately once per hour. Taylor et al. suggested switching to underpants made from an unfamiliar material during prescribed no-underpants times if nudity is not acceptable or feasible in a given setting. The investigators noted both that the duration of nudity decreased during each consecutive session and that the overall duration of nudity was not more than the participant’s estimated time out of clothing while cleaning up accidents prior to intervention.

Once urination reliably occurs in the toilet, it is possible that shaping may be applied to increase the duration of continent voids. Lomas Mevers et al. (2018) attempted to use a percentile schedule of reinforcement, in which the criterion for reinforcement of voids is recalculated with each successive void to shape differentially longer voids. However, only partial continence was reported at the end of the study (65% of voids occurred in the toilet) for the participant who received this modification. Croteau et al. (2022) describe a relatively simpler method for increasing urine output: when investigators observed that immediate reinforcer delivery terminated the flow of urine, they modified their procedure to wait until the urine stream was complete before delivering reinforcers.

##### Provide Water Prompts

One study described a procedure to elicit continent urination as a prerequisite to delivering reinforcement and establishing stimulus control over elimination in the toilet. To address a decrease in continent urination for their participant, Hagopian et al. (1993) poured a small amount of lukewarm water over the boy’s genitals for 3–5 s while he was seated on the toilet. When this procedure was added to BATT along with contingencies designed to reduce SIB (described under the previous barrier), continent urination increased. Hagopian et al. hypothesized that water prompting may have elicited urination, thus providing opportunities for reinforcement. Because the water prompt was not faded, it is unknown whether urine continence was achieved under naturally occurring stimulus conditions.

#### Apply a Negative Reinforcement Contingency for Toilet Sits

Four studies in this review detailed the use of negative reinforcement contingencies.

In one example, when a child would not use the toilet at school in the same way he did at home, Heyward (1988) initially designed a shaping and fading procedure to transfer stimulus control over continent urination from proximity of the child’s mother to proximity of a school classroom assistant. However, the child stopped urinating on the toilet as soon as his mother was no longer visible. Heyward then introduced a negative reinforcement procedure. The child was given 1.5 pints of liquid to drink at home without access to his home toilet. Upon arriving to school, the child sat on the toilet until he urinated. Within 1 day, the latency to urination decreased from 2.75 hr to less than 30 min. The child’s toileting schedule was later adjusted to match his classmates, and low latencies to urination maintained for at least 4 years.

In a second empirical demonstration of negative reinforcement techniques, Luiselli (2007) described a case study in which a child had never urinated in a toilet despite a history of scheduled 3-min toilet sits. First, the investigator presented a series of positive reinforcement contingencies illustrated on cards. The child continued to withhold urine on the toilet. Luiselli then introduced a negative reinforcement contingency by replacing the 3-min scheduled sit with sitting either until urination occurred or until 20 min elapsed, whichever came first. If the child did not urinate within 20 min, he was allowed to leave the toilet for 10 min and then return to sit for another 20 min until he urinated. This strategy was similar to the negative reinforcement procedure designed by Azrin and Foxx (1971) except that Luiselli ceased scheduled sits after one successful continent void each day.

#### Increase Opportunities for Positive Reinforcement of Continent Voids

Two studies portrayed increases in positive reinforcement contingencies to resolve urine retention. Luiselli (1997) described a toileting baseline that involved a negative reinforcement contingency applied to two daily toilet visits which was unsuccessful because the participant never urinated in the school toilet despite having used a toilet at home. The intervention included one training session per school day to minimize toileting demands. In contrast to Luiselli (2007), the training session was scheduled at the end of each day with the notion that the participant would be more likely to urinate with a full bladder. A positive reinforcement contingency (drinking water from a preferred container) was added to the negative reinforcement contingency, and the participant began to urinate on the toilet and experience both contingencies.

Post and Kirkpatrick (2004) initially elected to prompt toileting opportunities according to the natural schedule of incontinent voids observed in baseline. However, they observed that their participant urinated right after his training pants were reapplied following a sit without urination. Investigators implemented a regular 30-min sit schedule with 20-min prescribed sits, thus providing more opportunities for reinforcement of continent voids across the day. The sit schedule was systematically faded after 3 consecutive days with 80% continent voids. Later, a negative reinforcement contingency was added to each sit.

### How Can Practitioners Modify BATT for Children Who Have Ongoing Accidents after Reinforcement of Continent Urination?

Sometimes accidents occur despite frequent successful continent voids with contingent reinforcement and other elements of BATT. When accounting for the contrast between behavior and the known effects of reinforcement, it is possible that the reinforcer available for producing urine in the toilet is not as powerful as the reinforcement history for accidents or that the reinforcer is not applied contingently. However, assuming that reinforcer efficacy has been confirmed and procedural integrity is intact, research indicates the following modifications to BATT procedures under conditions of ongoing, or recurrent, accidents.

#### Enhance the Salience of Accidents

Recurrent accidents may signal a failure to establish continent urination as a discriminated operant. Thus, some researchers have explored methods to facilitate discrimination by enhancing the salience of accidents as detailed below.

##### Review Programmed Consequences

Although ethical precautions are warranted on a case-by-case basis, the addition of effortful requirements following accidents may result in behavior change. Lomas Mevers et al. (2018) added positive practice overcorrection as a remedial strategy for children who continued to have accidents after an initial BATT package without overcorrection. Of the 11 participants who met criteria for positive practice, five were reported to be continent at follow-up. Lomas Mevers et al. suggested that these mixed results might indicate either that positive practice was not sufficiently aversive to serve as a punisher or that positive practice might have functioned as a reinforcer for some participants.

##### Use a Urine Alarm or Implement Dry Pants Checks

A urine alarm alerts both the trainer and the child to the occurrence of accidents. For the trainer, the urine alarm permits immediate detection of accidents and immediate delivery of consequences. For the child, the alarm may increase awareness of the urge to urinate as well as occasion the engagement of muscles that control urine flow (Friman, 2010). Modern urine alarm technology includes wireless alerts for both learner and therapist as well as disposable sensors (e.g., Mruzek et al., 2019). A urine alarm was used in three studies that met criteria for this review (LeBlanc et al., 2005; Lomas Mevers et al., 2018; Taylor et al., 1994). Urine alarms have been proven efficacious across participants with varying skill levels (e.g., Lancioni & Markus, 1999), as a standalone treatment (Friman & Vollmer, 1995), and as components of comprehensive treatment packages (e.g., Azrin & Foxx 1971; LeBlanc et al., 2005). However, the literature also includes studies in which BATT variations with urine alarms were not effective (e.g., Didden et al., 2001; Lomas Mevers et al., 2018). In a review of the use of urine alarms, Levato et al. (2016) cited a lack of understanding of the behavioral mechanism that mediates improvement in toileting as well as insufficient overall evidence of the efficacy and effectiveness of urine alarms for treating incontinence.

Practitioners may incorporate a low-tech alternative to urine alarms such as a dry pants check. For example, Smith (1979) reported positive results when dry pants checks were implemented with fluid loading, scheduled toileting, and reinforcement of continent voids. Although six studies meeting criteria for inclusion in this review included dry pants checks as a treatment component, none provided within-subject comparisons of BATT with and without dry pants checks.

##### Exchange Diapers for Underpants

It is reasonable to conclude that the effects of accidents (e.g., wet pants) are more salient for children in underpants compared to an absorbent diaper. The discomfort of wet pants contingent on accidents may sometimes function as an automatic punisher, decreasing the likelihood of future accidents. Nine studies meeting criteria for inclusion in this review illustrate support for removal of diapers as a component of BATT. Cagliani et al. (2021) exchanged diapers for underpants as a component of a toileting baseline phase, along with a 90-min toileting schedule, graduated guidance following accidents, and differential reinforcement for continent voids. Following these simple manipulations, one participant’s accidents decreased to zero. It should be noted that the three remaining participants in this study required additional intervention components to achieve continence. Research beyond the scope of this review supports the removal of diapers for nondisabled children and adults with disabilities (e.g., Greer et al., 2016; Tarbox et al., 2004; Simon & Thompson, 2006).

A fading method may be an alternative to outright withdrawal of diapers. Ricciardi and Luiselli (2003) observed an increase in accidents for a participant whose incontinence appeared to be maintained by socially mediated consequences. Experimenters revised their procedure such that the participant wore a diaper throughout each day except for a 30-min interval that was randomly scheduled, during which he wore underpants. After the participant refrained from accidents during this 30-min interval for multiple days, the duration of the underpants interval was systematically increased.

#### Revise the Toileting Schedule to Provide More Opportunities for Reinforcement of Continent Voids

Four studies detailed increases in prescribed toilet sits as a method of decreasing accidents. For example, Richmond (1983) described accidents that persisted when children sat on the toilet each hour and received praise for continent urination. When the inter-sit interval (i.e., programmed duration of time away from the toilet) was reduced to 15 min during BATT, the investigator observed an immediate decrease in toileting accidents. The sit schedule was successfully faded over a period of 4 weeks to one sit every 2 hr. The denser toileting schedule might have increased the likelihood that urination would occur and be reinforced during toilet sits. However, simple correction for accidents (i.e., reprimands and instructions) was added at the same time as the schedule manipulation, so it is unclear whether a decrease in the inter-sit interval alone would be effective in this case.

Two recent publications provide guidance for data-based decision-making related to the sit schedule. Perez et al. (2020) implemented a denser sit schedule for two participants who did not become continent with package consisting of exchanging diapers for underpants, a 3-min toilet sit scheduled every 30 min, a dry pants check, and differential reinforcement for dry pants and in-toilet urination. The denser schedule (toilet sits every 15 min) was initially implemented during the time when accidents were observed most frequently and later extended across the day. A toddler potty chair was introduced to minimize time spent making the transition as a function of more frequent bathroom visits. One participant conformed to the denser sit schedule after the potty chair was implemented and their percentage of continent voids increased, but the second participant required a more comprehensive training solution. Cocchiola et al. (2012) moved one participant from a 30-min sit schedule to a 15-min sit schedule from 11:15 am to 12:30 pm as a BATT modification after observing a higher rate of accidents during this specific window of time. The 30-min sit schedule was in place during the remaining training hours each day, and the participant met the mastery criterion of 100% continent voids on a 2-hr schedule after 32 training days.

When a toileting schedule is revised to include shorter inter-sit intervals, researchers and practitioners must plan to efficiently fade the sit schedule so that the child may resume their regular activities. Fading criteria and duration varied across the included studies that modified sit schedules. For example, Richmond (1983) faded the toilet sit schedule over a month-long period. LeBlanc et al. (2005) faded a toilet sit schedule from prescribed sits every 5 min to zero scheduled sits over a period ranging from 9-23 days across three participants. It should be note that in the LeBlanc et al. study, one participant had not urinated as expected on the first day. Her sit schedule was held in place rather than faded until she successfully voided on the toilet. In a different approach to fading, Cicero and Pfadt (2002) removed their 30-min toilet sit schedule the first day after a participant’s unprompted initiation was followed by continent urination. Practitioners may consider conducting intermittent probes of the terminal schedule to potentially shorten the duration of training and the labor associated with schedule thinning (e.g., Perez et al., 2020).

Finally, adding a supplemental toilet sit is an alternative to setting one inter-sit interval for the entire training day. Cagliani et al. (2021) observed ongoing morning accidents despite the participant’s experiences with positive reinforcement, so they scheduled an extra bathroom trip to occur in the morning without requiring more frequent toileting visits throughout the day. The percentage of voids occurring in the toilet increased to 100%.

#### Monitor Fluid Intake

Although it is important for children to be well-hydrated, the additional fluids offered during toilet training may result in increased accidents. Both Cagliani et al. (2021) and Doan and Toussaint (2016) observed increases in accidents correlated with the provision of additional fluids. Doan and Toussaint reported eventual decreases in accidents, and Cagliani et al. discontinued additional fluids when accidents did not resolve after several sessions. Increased hydration occurring outside of the training setting may also need to be considered: Chung (2007) observed that accidents occurred after a participant swallowed excessive amounts of water while swimming.

#### Use a Urine Target

Sometimes voids occur on an ongoing basis near (but not in) the toilet. This type of toileting accident may be related to either a skill deficit or access to reinforcers associated with urinating onto the seat or floor around the toilet. Siegel (1977) placed a visual target in the toilet to reduce accidents with three participants. Prior to the intervention, participants regularly urinated on the seats, walls, and floors of toilet stalls. During treatment sessions, Siegel prescribed increased fluid intake for participants and a visual target was placed in the toilet. Participants began to direct their urine streams toward the target, resulting in fewer accidents. When the target was removed, accidents increased. The target was replaced, and accidents decreased again. In the final phase, participants were instructed to hit the target, resulting in near-zero rates of accidents. Siegel reported that participants appeared to enjoy trying to hit the target.

### How Can Practitioners Modify BATT with Children Who Self-Initiate Too Rarely or Too Often?

It would be ideal for well-hydrated children to learn to identify the urge to urinate (i.e., proprioceptive stimuli), initiate an independent toilet request, and proceed to the toilet to void. Multiple studies have included prompts for participants to request the toilet as a component of BATT (e.g., Cagliani et al., 2021; Cicero & Pfadt, 2002; LeBlanc et al., 2005, Perez et al., 2020). Effects on self-initiations have been mixed. For example, Kroeger and Sorensen (2010) reported that 100% of toileting events were self-initiated for both participants at the end of their investigation. Croteau et al. (2022), by contrast, observed a decrease in self-initiations from 1.5 per day in baseline and initial treatment to 0 by the end of treatment for one participant and little to no self-initiations from the other three participants.

Differences in the outcomes of studies that addressed self-initiations during BATT may be related to varying definitions of self-initiation. Azrin and Foxx (1971) defined self-initiation as independent toilet approach (p. 93). Cicero and Pfadt (2002) redefined self-initiations as the child requesting the toilet in the absence of prompting. Cicero and Pfadt’s definition may be more practical for children with disabilities who require caregiver supervision and must ask to use the toilet across settings (e.g., school, community). It should be noted that six of the seven most recently published studies meeting criteria for inclusion in this review included unique definitions for self-initiations. These definitions encompassed a range of self-initiation topographies, and some but not all definitions included that participants should urinate after requesting and accessing the bathroom. Once established, self-initiations may become problematic if they occur too frequently (i.e., more often than the child needs to empty their bladder).

#### Teach the Child to Respond Independently to Supplemental Cues

If a client does not demonstrate self-initiations under conditions of a full bladder and a history of contingent access to the bathroom, it may be helpful to introduce supplemental prompts while fading adult prompting. Luiselli (1987) observed a failure to teach self-initiations with a toileting schedule, restitutional overcorrection (washing clothes), and reinforcement for completing a toileting routine. The investigator gave the participant a wristwatch and instructed her to request the bathroom within specified time intervals. Reinforcement was contingent on the emission of toilet requests within the correct interval followed by continent urination. Self-initiated toileting increased from near-zero to approximately 14 instances per week.

#### Discontinue Prompts to Toilet

LeBlanc et al. (2005) suggested that frequent toilet prompts may inhibit self-initiations due to insufficient motivation (i.e., children are less likely to experience a full bladder). Thus, they implemented a sit schedule that was rapidly faded across days and removed when the participant reached 80% success with a 4-hr inter-sit interval across 2 consecutive days. In total, 11 studies included in this review included progressive sit schedules with fading. As a component of their toilet training package, Lomas Mevers et al. (2018) included a toilet sit schedule that increased time away from the toilet in 5 to 15 min increments. The inter-sit interval was increased or decreased based on continent or incontinent voids. If participants progressed through the schedule up to 60 min away from the toilet without the emergence of self-initiations, Lomas Mevers et al. discontinued the sit schedule and therefore relied upon self-initiations. Investigators reported that this strategy was associated with sustained continence (100% continence on the last day for four participants; 67% continence for one participant), although data on self-initiations were not reported. By contrast, continence decreased for at least one participant when Perez et al. (2020) removed the sit schedule, and only 3 of 11 participants were reported to self-initiate toilet requests at the conclusion of the study. Seven participants continued to require a 120-min prompted sit schedule, and 1 participant required more intensive training beyond the methods employed in the Perez et al. study.

#### Implement Functional Assessment to Identify Potential Maintaining Reinforcers for Requests

Research indicates that toilet requests may serve multiple functions, indicated when self-initiations to toilet are not consistently followed by voids. For example, Perez et al. (2020) reported correspondence values between self-initiations and subsequent continent urination for 11 participants post-mastery of targeted toileting skills. The values ranged from 50% or lower (3 participants) to 100% (2 participants), suggesting that, in some cases, self-initiations may have been maintained by escape from instructions or access to restricted items. Ewing et al. (2001) conducted a functional analysis of perseverative toilet requests for one participant and found that rates were differentially higher in the attention condition. These findings suggested that the toilet request was maintained by access to attention rather than access to the opportunity to void. 

#### Identify and Fade Unnecessary Training Components

Following the establishment of self-initiated toileting, toilet requests may occur at a higher than expected rate. After biological influences are ruled out, one plausible cause of excessive self-initiations is the ongoing presence of training components which are no longer required for skill acquisition. For example, Cicero and Pfadt (2002) successfully trained self-initiated toileting with three participants as part of an initial training package that included scheduled prompts to request the toilet followed by toilet sits and reinforcement for continent voids. However, they observed continuously increasing rates of toilet requests (i.e., as many as 17 requests per school day). After fading training components such as time spent in the bathroom environment, fluid loading, and reinforcers for continent voids, requests decreased to a rate of 1–2 per day with accidents remaining at 0.

#### Implement a Multiple Schedule of Reinforcement

Lomas Mevers et al. (2018) described the use of a multiple schedule of reinforcement to treat excessive requests. Self-initiations for the bathroom were reinforced during a 5-min period each hour. The therapist wore a bracelet to indicate when the reinforcement component of the schedule was in effect, and access to the bathroom was withheld for requests that occurred in the 55-min schedule component. By the last day of training, continence for this participant was reported to be 100%, although it is unclear whether all voids were self-initiated.

## Discussion

The literature on BATT includes many individualized strategies for resolving barriers to daytime urine training for children with disabilities. These solutions may prove useful to practitioners when research-supported BATT packages appear ineffective and underlying medical conditions and problems of adherence or fidelity have been ruled out as possible explanations. The current article was intended to help practitioners navigate barriers that may arise during toilet training. It is important to reiterate the value of medical collaboration prior to and during toilet training for children with disabilities. Practitioners should be aware of general medical and physiological considerations with implications for continence (e.g., constipation, infections, or diabetes may cause recurrent accidents; urine retention may result from dehydration or infections) so they do not modify BATT when a medical consultation is warranted. Practitioners should also be aware of the potential for medical concerns to arise during BATT such as overhydration (Thompson & Hanson, 1983).

Some limitations within the current literature, including results of this review, may make it difficult for practitioners to access barrier solutions. For example, descriptions of BATT modifications are often insufficiently technological (e.g., fluid restriction is prescribed but the amount of fluid presented is not specified nor contrasted with typical fluid consumption). Investigators conducting future research should ensure that participant characteristics and procedures are clearly described to facilitate replication. Practitioners who routinely provide toilet training services and have access to research-related resources (e.g., ethical review committees) are uniquely positioned to conduct these studies as they follow the EBP process and evaluate toilet training with their clients. For example, criteria for establishing a sit schedule and subsequent fading should be explored empirically to inform practice. Comparative analyses will also be useful (e.g., inter-sit interval values, efficiency of BATT with and without dry pants checks). Bacotti et al. (2023) provide additional recommendations for future research to advance toilet-training technology. Practitioners should remain in contact with the literature as future studies are published and update their practice to help more of their clients achieve continence.

A second limitation of the results of this review is that multiple modifications were often presented to address a single barrier (e.g., DRA, decreasing sit schedules, and eliminating overcorrection may all be incorporated to address severe problem behavior during toilet training), and some studies that addressed barriers did not include data specific to barrier remediation (e.g., Duker et al., 2001, described problem behavior but did not report occurrence data). Thus, the extent to which individual modifications are necessary or even effective in remedying specific barriers remains unknown. Smith (2012) cited this uncertainty as a consistent problem with research on treatment packages. To illustrate, Johnson et al. (2021) evaluated the presence or absence of 14 BATT intervention components across 57 studies and showed that no single study included all 14 components. The most commonly observed component was diaper removal, followed by punishment, increased fluids, and differential reinforcement of dry pants checks. Friman (2010) noted that some cases of incontinence may be resolved with a minimal number of components. However, he recommended for practitioners to prescribe as many treatment components as caregivers are willing to implement, and as children are willing to complete, to achieve the greatest likelihood of treatment success.

A third factor limiting the strength of recommendations in this review is that the same barrier solution was rarely applied with multiple participants. Instead, they were often designed to address an idiosyncratic issue within a specific context. One exception is the consecutive case series analysis published by Lomas Mevers et al. (2018). In this study, as many as 11 participants experienced the same BATT modification (positive practice to address recurrent accidents). Nonetheless, researchers are yet to demonstrate functional relations between a specific barrier and the BATT modification that is efficacious for addressing it. One minor consolation is that the potential solutions described in past research are rooted in behavior-analytic principles (e.g., reinforcement), and procedures based on these principles have been applied to influence a variety of socially important behaviors in children with disabilities (e.g., Gerow et al., 2018). Rather than a proverbial “collection of tricks” (Baer et al., 1968, p. 96), these barrier solutions can be viewed as options within a conceptually systematic approach to teaching urine continence.

Findings of this review raise several barrier-specific questions worthy of closer examination. Research suggests that overcorrection, a relatively common component of BATT, may both contribute to barriers (e.g., increased problem behavior) and resolve barriers (e.g., decreased accidents). Findings of this review suggest that practitioners should weigh the social and practical importance of a client achieving urine continence against the feasibility of maintaining a safe toileting environment for clients and implementers. As demonstrated by Hagopian et al. (1993), treatments that result in decreased problem behavior may also result in diminished continence, and treatments that result in improved continence may be associated with higher rates of problem behavior due to a change in the magnitude of demands (e.g., during positive practice) or other aversive stimulation. For example, after implementing a modified version of BATT that did not include overcorrection, Chung (2007) observed ongoing accidents in his participant in addition to frequent continent urination. Chung postulated that overcorrection, although potentially not essential for learning correct toileting behavior, may be important for decreasing accidents. As Hanney et al. (2013) remarked, accidents are always negatively reinforced by bladder relief. Positive reinforcers for continent urination may be insufficient to produce differential reinforcement effects, offering a conceptual rationale for the use of added consequences to reduce accidents when the use of such procedures meets all necessary ethical requirements.

Practitioners should note that overcorrection functions as a punishment procedure when its contingent delivery results in a decreased frequency of the response it follows. In their review of toilet training intensity, Johnson et al. (2021) found that punishment was a component of 60% (*n* = 33) of studies that met criteria for inclusion. In addition to overcorrection, reviewed studies included procedures that may function as contingent punishment including response restriction (e.g., limiting the participant’s space to within a small distance from the toilet; Azrin & Foxx, 1971; Duker et al., 2001), response blocking (i.e., blocking attempts to engage in behavior other than targeted toileting responses; Chung, 2007; Duker et al., 2001), and contingent exercise (applied following aggression or SIB that occurred during toileting overcorrection; Foxx & Garito, 2007). Given ethical standards which apply to the use of punishment, we elected to refrain from describing these interventions in detail as barrier solutions. At the time of this review, the *Code* (BACB, 2020) requires that restrictive or punishment-based procedures are used “only after demonstrating that desired results have not been obtained using less intrusive means, or when it is determined by an existing intervention team that the risk of harm to the client outweighs the risk associated with the behavior-change intervention” (p. 12). Of course, there may be circumstances in which these procedures are warranted. The EBP process can help to guide decision making among practitioners, clients, and community members (e.g., caregivers, implementers). Practitioners should be aware of local laws, licensure regulations, and organizational policies when proceeding with these types of interventions.

Some of the studies in this review included relatively complex procedures such as the use of functional assessment to determine the conditions occasioning and maintaining toileting-related problem behavior (e.g., Dowdy et al., 2020; Flood & Luiselli, 2016; Foxx & Garito, 2007; Ricciardi & Luiselli, 2003). Functional analysis is best practice prior to developing function-based treatments for problem behavior (for a review, see Melanson & Fahmie, 2023), but past toileting research is limited in this respect. It is therefore unclear whether a functional analysis of interfering behavior during toileting is necessary. Brief observation of a toileting session may help practitioners to identify simple manipulations that resolve interfering behavior, such as shortening the duration of toilet sits to eliminate aggression that, in the past, produced termination of an extended sit. An interview may reveal likely reinforcers for problem behavior (e.g., escape, attention) that can be incorporated into differential reinforcement programs (e.g., Hanley, 2012). The toileting environment could be made less generally aversive with a “potty party” strategy in which snacks and preferred activities are provided noncontingently during extended toilet sits (e.g., reading and singing; Flora et al., 2020).

Barriers to self-initiation also merit further discussion. Studies described in this review are characterized by a lack of consistency in defining self-initiations to toilet as well as procedures used to achieve those outcomes. Practitioners should consider a client’s future settings when collaboratively setting goals for self-initiations (e.g., spontaneous walk to the bathroom versus verbal request). Selecting a communication modality or teaching procedure for teaching toileting requests is an opportunity for collaboration with service providers in the professions of speech-language pathology, alternative augmentative communication, or assistive technology. After target responses and teaching procedures have been identified, practitioners ought to ensure that toilet requests are contingently followed by toilet visits with reinforcers for continent urination. As Ewing et al. (2001) suggested, careful programming of reinforcers can help to ensure that participants learn the appropriate response–consequence relation. The practice of prompting toilet requests prior to a toilet visit is common based on studies reviewed herein. However, practitioners should consider the possibility that these procedures may be unlikely to establish self-initiations in the absence of adult prompts (i.e., prompt dependency; Clark & Green, 2004), nor may they establish self-initiations maintained by the desired reinforcers of bathroom access, bladder relief, and avoidance of any natural or programmed consequences of accidents.

Questions remain about what constitutes a barrier in need of a reviewed solution in practice. For example, practitioners may experience client noncompliance with BATT procedures as a potential barrier. It is important to remember that the *Code* (2020) requires behavior analysts to treat clients with “compassion, dignity, and respect” (p. 4). Therefore, when clients do not agree with participating in behavioral procedures, practitioners should adhere to assent-related responsibilities. The *Code* (BACB, 2020) defines assent as “Vocal or nonvocal verbal behavior that can be taken to indicate willingness to participate in research or behavioral services by individuals who cannot provide informed consent (e.g., because of age or intellectual impairments)” (p. 7). It can be difficult to obtain assent in clients with disabilities (Morris et al., 2021). However, when a child engages in problem behavior during toilet training or demonstrates other forms of counter-control in response to research-supported toilet training procedures, the practitioner must consider whether the child has withdrawn their assent from these procedures or goals and use their knowledge of ethical standards to collaboratively determine next steps.

Behavior analysts also have a responsibility to protect their clients from harm and prioritize goals consistent with the client’s best interests. In the event that continence is a high-priority goal, yet a child appears unwilling to assent to toilet training procedures, it is incumbent upon the behavior analyst to continue to engage in the EBP problem-solving process. For example, they can thoroughly examine and reduce the potential aversiveness of procedures, find ways to incorporate client choice and reinforcers, and evaluate other modifications that may improve the child’s overall experience while working toward continence. Recent literature includes examples of some ways that practitioners can incorporate parent input and choice in selecting BATT components and intensity (e.g., Dabney et al., 2023; Doan & Toussaint, 2016). In addition, given evidence of fear responses particular to the toileting context (e.g., Koegel et al., 2004; Luiselli, 1977), practitioners should be prepared to consult professionals with expertise in systematic desensitization and trauma-informed support (see Rajaraman et al., 2022) or to refer a client to alternative service providers if the nature or complexity of their needs falls outside the practitioner’s current scope of competence (Standard 1.05; BACB, 2020).

One factor that is likely to affect the acceptability of certain toileting goals and procedures for clients and families is the cultural and economic implications of those goals and procedures. Practitioners are encouraged to consider and respond to cultural aspects of toileting that are not presently well-represented in the literature. It is possible that some barrier solutions appropriate to one context are culturally inappropriate in another. For example, cultural values around privacy and modesty may influence whether a practitioner offers a toddler potty chair in a bathroom or classroom setting as well as how much clothing a client wears during training. The acceptability of teaching biologically male participants to stand versus sit when urinating may vary across cultures as well as for specific families within a given culture. Further, family expectations about the age at which training should start have been found to vary by biological sex, child developmental status, and familial economic resources (Howell et al., 2010). Toileting strategies, whether they are part of initial BATT or a barrier-specific modification, should only be implemented to the extent that they align with the social-cultural context as well as client and community member values.

Behavior analytic toilet training strategies based on Azrin and Foxx (1971) have been used across populations and settings for over half a century. Nonetheless, behavior analysts often modify BATT due to contextual constraints and individualized needs or response patterns. The toilet training literature houses a diverse array of these modifications to the original BATT components. It is our hope that strategies compiled here will equip practitioners to overcome barriers to urine continence with more of their clients. We also hope that some practitioners will be inspired to refine the existing pool of BATT modifications by developing, evaluating, and eventually disseminating conceptually-systematic solutions to barriers their clients encounter.

## Data Availability

Not applicable

## Declarations

### Publication history

This manuscript has not been previously published. This manuscript has not been or will not be submitted elsewhere during the review process.
